# Characterization of phytoconstituents and evaluation of antimicrobial activity of silver-extract nanoparticles synthesized from *Momordica charantia* fruit extract

**DOI:** 10.1186/s12906-017-1843-8

**Published:** 2017-06-26

**Authors:** Md. Mamun Or Rashid, Kazi Nahid Akhter, Jakir Ahmed Chowdhury, Foysal Hossen, Md. Saddam Hussain, Md. Tanvir Hossain

**Affiliations:** 1 Department of Pharmacy, Noakhali Science and Technology University, Sonapur, Noakhali, 3814, Bangladesh; 2 Department of Pharmaceutical Technology, University of Dhaka, Dhaka, 1000, Bangladesh; 3 Department of Microbiology, Noakhali Science and Technology University, Sonapur, Noakhali, 3814, Bangladesh; 4 Department of Applied Chemistry and Chemical Engineering, Noakhali Science and Technology University, Sonapur, Noakhali, 3814, Bangladesh

**Keywords:** Silver nanoparticles (AgNPs), Silver extract nanoparticles (Ag-extract-NPs), *M. charantia*, Antibacterial activity

## Abstract

**Background:**

Our present study was conducted to characterize the phytoconstituents present in the aqueous extract of *Momordica charantia* and evaluate the antimicrobial efficacy of silver-extract nanoparticles (Ag-Extract-NPs).

**Methods:**

Silver nanoparticles (AgNPs) were prepared by reducing AgNO_3;_ and NaBH_4_ served as reducing agent. After screening of phytochemicals; AgNPs and aqueous extract were mixed thoroughly and then coated by polyaniline. These NPs were characterized by using Visual inspection, UV spectroscopy, FTIR, SEM and TEM techniques. Antimicrobial activities were assessed against *Staphylococcus aureus*, *Salmonella typhi, Escherichia coli* and *Pseudomonas aeruginosa*.

**Results:**

Aqueous extract of *M. charantia* fruits contain alkaloid, phenol, saponin etc. UV–Vis spectrum showed strong absorption peak around 408 nm. The presence of –CH, −NH, −COOH etc. stretching in FTIR spectrum of Ag-Extract-NPs endorsed that AgNPs were successfully capped by bio-compounds. SEM and TEM result revealed that synthesized NPs had particle size 78.5–220 nm. Ag-Extract-NPs showed 34.6 ± 0.8 mm zone of inhibition against *E. coli* compared to 25.6 ± 0.5 mm for ciprofloxacin. Maximum zone of inhibition for Ag-Extract-NPs were 24.8 ± 0.7 mm, 26.4 ± 0.4 mm, 7.4 ± 0.4 mm for *S. aureus, P. aeruginosa* and *S. typhi*. We found that Ag-Extract-NPs have much better antibacterial efficacy than AgNPs and *M. charantia* extract has individually. It is also noticed that gram negative bacteria (except *S. typhi*) are more susceptible to Ag-Extract-NPs than gram positive bacteria.

**Conclusion:**

Ag-Extract-NPs showed strong antibacterial activity. In order to make a reliable stand for mankind, further study is needed to consider determining the actual biochemical pathway by which AgNPs-extracts exert their antimicrobial effect.

**Electronic supplementary material:**

The online version of this article (doi:10.1186/s12906-017-1843-8) contains supplementary material, which is available to authorized users.

## Background

Nanoparticle research has become an area of intense scientific interest due to a wide variety of potential applications in electronic, optical and biomedical fields [1, 2]. It is found that nanoparticles have the capability to fix, adsorb and carriage other compounds such as drugs, probes and proteins because of their large surface area [3].

Nowadays, the generation of antibiotic-resistant bacteria has become the greatest health challenges and serious concerns to be considered [4]. In recent times, nanoparticle technology got the pin point in the development of new antimicrobial agent which is successfully used against antibiotic-resistant bacterial strain. It is great to see in recent year various keyboards, wound dressings, textiles, and biomedical devices containing silver nanoparticles are generated which provide protection against bacteria through continuous release of low level of silver ions [5]. Numerous types of nanomaterials and their composites have been applied as antimicrobial agents such as titanium, copper, zinc, gold, and silver [6–9]. Despite some actual concernment, regarding their toxicity [10], AgNPs demonstrate very good antimicrobial activity against bacteria, viruses, and other eukaryotic microorganisms [11]. Several studies have afforded to explicate the biochemical pathway by which AgNPs pose their antibacterial activities [12–14]. It is supposed that large surface area of NPs and their capability to successfully release silver ions is the pin point to their antibacterial activity [15]. The discharge of ionic silver causes cell death through inactivation of vital bacterial enzymes, inhibition of DNA replication, and by rupturing the bacterial cytoplasmic membranes [12].

*Momordica charantia* commonly known as bitter gourd is a tropical and subtropical vine of the family Cucurbitaceae*,* widely grown in Asia, Africa and the Caribbean for its edible fruit, which is extremely bitter in taste [16]. Different parts of the plant are medicinally used to recover from diabetes and also used in following aspects such as antibilious, stomachic, emetic, anthelmintic agent, laxative, for the treatment of skin diseases, cough, wounds, gout, ulcer, respiratory diseases and rheumatism [17]. Present study is designed to characterize the phytoconstituents present in *M. charantia* and the antibacterial activity of *M. charantia* extract loaded AgNPs.

## Methods

### Materials collection

Fresh unripe fruits of *M. charantia* were collected from the local market of Noakhali, Bangladesh. Later, these were washed thoroughly by distilled water several times. The plant’s material was identified and authenticated by the National Harberium, Mirpur, Dhaka. The voucher specimen no. was DACB 42292. Silver Nitrate (MERCK, Germany), NaBH_4_ (LOBA CHEMIE), aniline and all other chemicals used in this experiment were analytical grades and collected from the laboratory of the Department of Pharmacy, Noakhali Science and Technology University.

### Extract preparation

About 200 g fresh bitter gourds were collected from Noakhali district of Bangladesh. The fruits were washed thoroughly with double distilled water, cut into fine pieces and boiled with 1000 mL distilled water for about 1 h. The extract was cooled at room temperature and filtered by cotton and Whatman filter paper. Prepared clear extract was stored in refrigerator at 4 °C for further use.

### Phytochemical screening

The extract solution was used for preliminary screening of phytochemical such as alkaloids, tannin, phenolic compound, saponin, glycoside, flavonoid, protein, reducing sugar, phytosterol, gum and mucilage etc. by using standard procedures described by Mbaebie et al. [18].

### Synthesis of silver nanoparticles

As described in our previous paper [16], Ag nanoparticles were prepared according to the recipe described in the literature of Creighton, 1979 with slight modification [19]. Briefly, 34 mg (1.0 X 10^−3^ M) AgNO3 was added with 200 mL double distilled water and 45.4 mg (2.0 X 10^−3^ M) NaBH_4_ was added with 600 mL double distilled water. After chilling the solutions at 0 °C, mixing was done with continuous stirring for 1 h. Due to mixing, Ag ions were reduced and formed mono-dispersed nanoparticles. At this point this solution of Ag nanoparticles was so stable that it did not change color for a long time without any stabilizing agent. Solution was concentrated 10 times using a rotary vacuum evaporator as particle concentration of the solution was very low.

### Preparation of Ag-extract-NPs

Extract and Ag nanoparticles solutions were mixed continuously for 30 min by magnetic stirrer and then kept for 2 h. Due to mixing, silver nanoparticles were loaded with extract to form Ag-Extract-NPs. This uncoated Ag-Extract-NPs need to be coated for making it more stable.

### Coating of Ag-extract-NPs by polyaniline

Coating on the Extract-AgNPs were done according to the Rashid et al. [20]. 2.7 g aniline was dissolved into 300 mL deionized water. At the same time, 68 mL (6%) of H_2_O_2_ was also prepared. These two solutions were added slowly with uncoated Ag-Extract-NPs at room temperature for 30 min. The mixture was allowed to stir continuously for 23 h. The final product was filtered, washed by distilled water and then dried at room temperature. Finally, Ag-Extract-NPs were ready for further analysis (antimicrobial activity).

### Characterization studies

Prepared AgNPs need to be characterized for confirming those as nanoparticles. Color changes during experiment endorsed the formation of nanoparticles. Reduction of pure silver ions was monitored by measuring UV-Vis spectra of the reaction mixture. UV-Vis spectra were measured using Hitachi, U-2800 spectrophotometer model (UV- Visible double beam). For characterizing, absorption spectra of the samples were taken 200 to 700 nm. FTIR spectra (IR Prestige-21, Shimadzu) were also performed and recorded in the range of 4000–400 cm^−1^ at a resolution of 4 cm^−1^. Size, shape and morphology of nanoparicles were determined by using Scanning Electron Microscopy (2600SN Hitachi, Japan) from BCSIR, Bangladesh and Transmission Electron Microscopy (HITACHI H-700, Japan) images were taken from the Departmental laboratory of Applied Chemistry and Biochemistry, Kumamoto University, Japan.

### Test microorganisms

Authentic pure cultures of *Staphylococcus aureus* ATCC 25923, *Salmonella typhi* ATCC 14028, *Escherichia coli* ATCC 25922 and *Pseudomonas aeruginosa* ATCC 27853 were obtained from the department of microbiology, NSTU, Bangladesh.

### Antimicrobial activity using Kirby-Bauer’s disc diffusion method

Antibacterial activities of the Ag-Extract-NPs were determined using a modified Kirby-Bauer’s disc diffusion method [21]. Solutions of known concentration of the test samples were made by dissolving a measured amount of the samples in a calculated amount of solvents. Dried and sterilized filtered paper discs (6 mm diameter) were then impregnated with known amounts of the test substances using micropipette. The discs containing tested material were placed on Muller-Hinton agar media which were inoculated with test microorganisms previously. Ciprofloxacin discs (standard) and blank discs (impregnated with solvents) were used as positive and negative control. Agar plates were then allowed to stand at 4 °C for 2 h before incubation. During this time dried discs can absorb water from the surrounding media and then test materials were dissolved and diffused out of the sample disc. Plates were incubated at 37 °C for 24 h to allow maximum growth of the organisms. The antimicrobial activities of the test agents were determined by measuring of zone of inhibition expressed in millimeter.

### Statistical analysis

Data were analyzed using one way ANOVA tests (SPSS software, version-20) followed by Dennett’s t-tests. *p < 0.05, **p < 0.01, ***p < 0.005 were considered significant.

## Result and discussion

### Phytochemical composition of *M. charantia*

Phytochemical screening of aqueous extract of *M. charantia* is shown in Table 1. Result revealed that the extract contains remarkable amount of alkaloid, phenolic and saponin compounds. In addition, we also found the presence of tannin, glycoside, protein, reducing sugar etc. in the aqueous extract of *M. charantia*.

**Table 1:** Phytochemical analysis of aqueous extract of *M. charantia* fruits

Phytochemical	Test name	Aqueous extract of *M. charantia*
Alkaloid	Meyer Test	+
	Wagner Test	++
Phenolic	Ferric Chloride Test	+
	Lead Acetate Test	++
Tanins	Ferric Chloride Test	+
	Potassium Dicromate Test	−
Saponin	Froth’s Test	++
Glycoside	Keller Kilani Test	+
Protein	Xanthroprotic Test	+
Reducing Sugar	Fehling’s Test	+
Phytosterol	Liebermann-Burchard’s Test	_

Here, ++ = Moderate; + = Normal Concentration; − = Absent

### Characterization of AgNPs

#### Assessment of AgNPs through visual assessment

Prepared silver nanoparticles were evaluated first by visual assessment. Due to reaction within 20 min, color of the mixture turned into dark brown from its initial brownish yellow color. Such color change indicated the formation of silver nanoparticles due to the reduction of AgNO_3_.

#### Assessment of AgNPs by UV spectrum

**Fig. 1:**
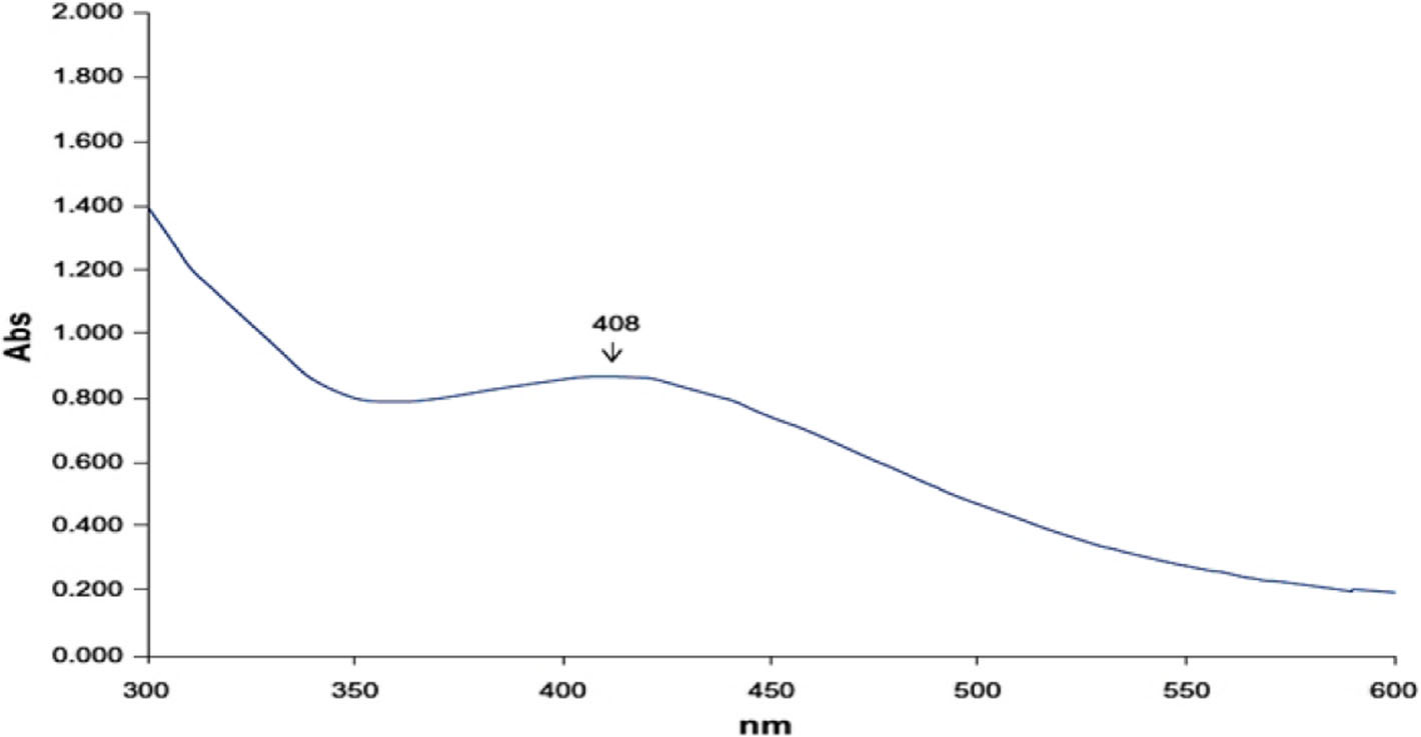
UV-Vis spectrum of AgNPs

The reaction mixture was allowed to UV–Vis spectrum at different wavelengths ranging from 300 to 700 nm. Strong absorption peak centering at approx. 408 nm (Fig. 1) was found, which indicated the formation of AgNPs.

#### Assessment of Ag-extract-NPs through FTIR spectra

**Fig. 2:**
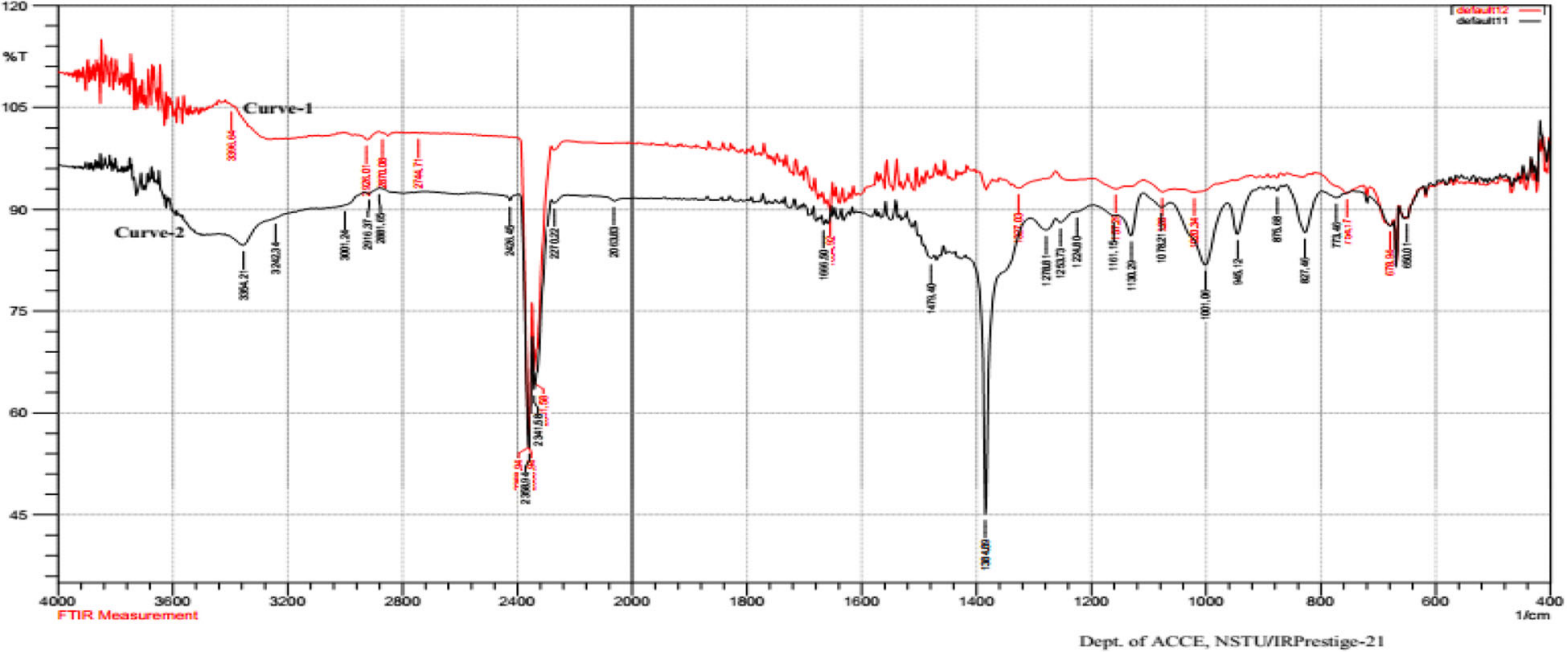
FTIR spectrum of *M. charantia* extract (Curve-1) and Ag-Extract-NPs (Curve-2)

The FTIR spectra were used for confirming the possibility of capping by *M. charantia* extract on AgNPs. The possible functional groups of leaf extract involved in coating nanoparticle are identified by FTIR analysis which is shown in Fig. 2. The intense absorption peaks at 2926.01 cm^−1^ and 2870.08 cm^−1^ (curve-1) and 2916.37 cm^−1^ (curve-2) correspond to –C-H stretching of alkane. The band observed at 3354.21 cm^−1^ (curve-2) is for N-H stretching of primary amine. The band observed at 2358.94 cm^−1^, 2341.58 cm^−1^ (curve-1) and 2426.45 cm^−1^, 2358.94 cm^−1^, 2341.58 cm^−1^ (curve-2) represent the presence of –COOH group which may exist in the fruit extract of *M. charantia* and Ag-Extract-NPs. In addition, stretching for alkene (−C = C-) was found at 1654.92 cm^−1^ (curve-1) and 1666.50 cm^−1^ (curve-2). The peak found at 1470.50 cm^−1^ (curve-1) and 1479.40 cm^−1^ (curve-2) correspond to –CH scissoring.

**Fig. 3:**
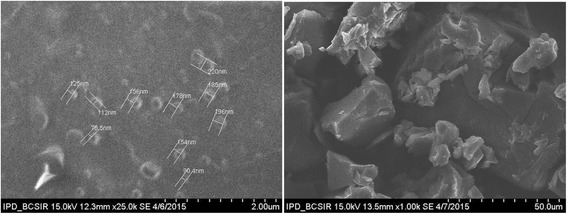
SEM image of Ag-Extract-NPs

**Fig. 4:**
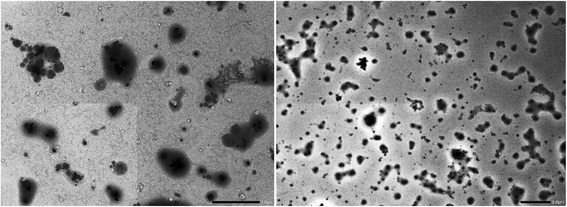
TEM image of Ag-Extract-NPs

**Fig. 5:**
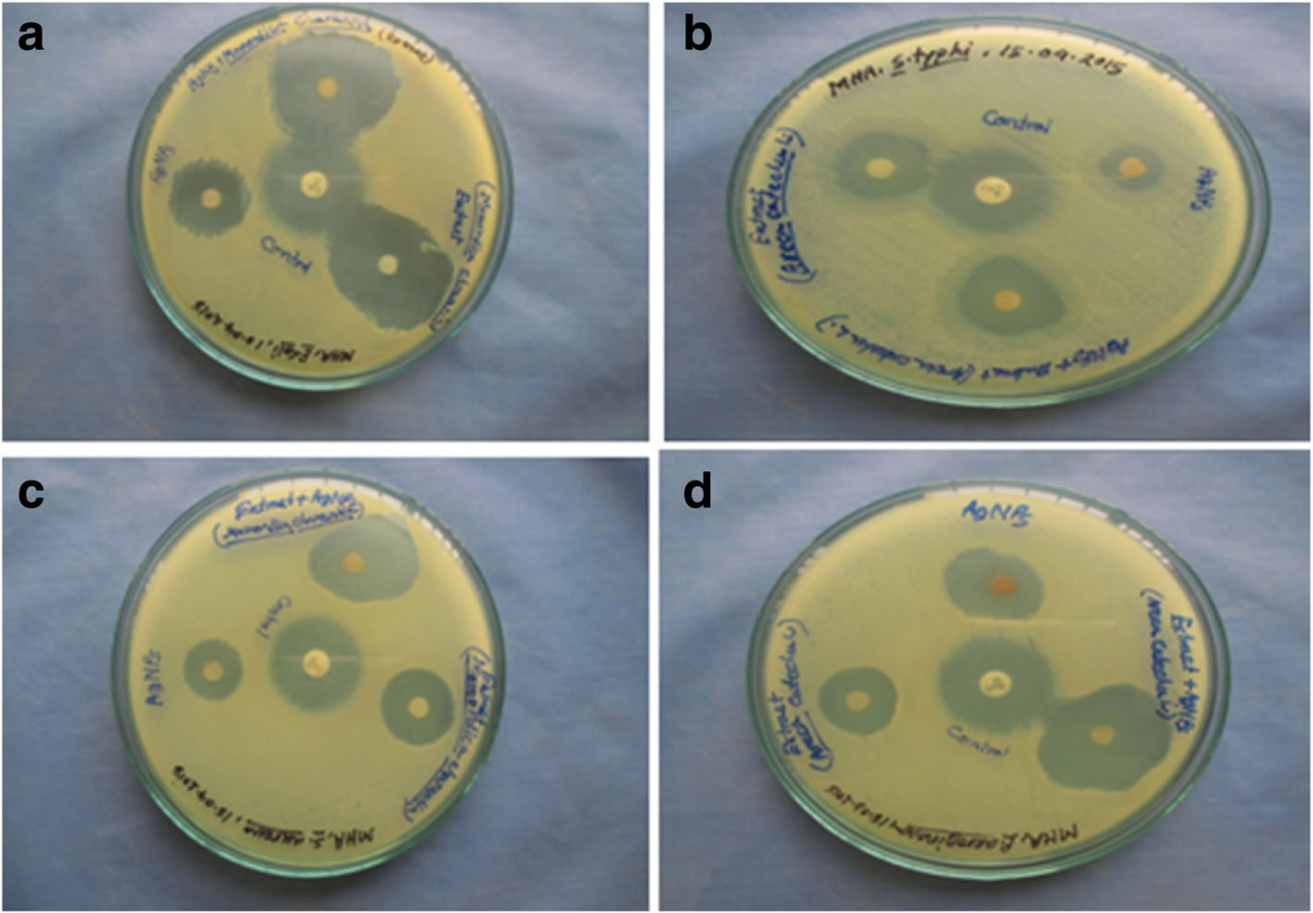
Antibacterial activities of *M. charantia*, AgNPs, Ag-Extract-NPs against- **a** *E. coli* **b** *S. typhi* **c** *S. aureus* **d** *P. aeruginosa*

The band observed at 1384.89 cm^−1^ (curve-2) represent N = O stretching of nitro groups and 1253.73 cm^−1^ (curve-2) indicate the presence of alkyl halide or alcohol group in fruit extract coated on nanoparticles. The arising of functional groups in FTIR spectrum indicates proper coating of leaf extract on silver nanoparticles. The bands at 1130.29 cm^−1^ (curve-2) denoted -C-H stretching vibration of ester. Beside, C-O stretch occurred at 1001.01 cm^−1^. The bands at 754.17 cm^-1,^ 711.50 cm^−1^, 678.94 cm^−1^, 670.25 cm^−1^ (curve-1) and 827 cm^−1^, 678.94 cm^−1^, 670.25 cm^−1^ represent the ortho substituted and mono substituted aromatic stretching respectively. The FTIR results imply that aqueous fruit extract of *M. charantia* was successfully capped on AgNPs and Ag-Extract-NPs were successfully synthesized.

#### Assessment of Ag-extract-NPs by SEM and TEM imaging

SEM and TEM analysis gave us information about structural morphology, size and shape of the synthesized Ag-Extract-NPs (Figs. 3 and 4). It was found that the Ag-Extract-NPs were roughly circular in most of the cases; however in few cases they were irregular. The diameter of these nanoparticles lied between 78.5 to 100 nm in most cases, however few particles were larger than average diameter. The nanoparticles were not in direct contact with each other even within the aggregates, indicating stabilization of the nanoparticles by a capping agent. The larger particles found in SEM measurements were probably due to the aggregation of the smaller ones.

#### Assessment of in-vitro antibacterial activity

Antibacterial activities of aqueous extract of *M. charantia*, AgNPs, Ag-Extract-NPs were tested against both gram positive and negative bacteria. The zone of inhibition due to their antibacterial efficacy were measured and shown in Table 2 , Fig. 5 (see details in Additional file 1: Table S1). It was found that maximum zone of inhibition produced by *M. charantia* extract, AgNPs and Ag-Extract-NPs were 16.6 ± 0.5 mm, 14.4 ± 0.5 mm and 24.8 ± 0.7 mm respectively when tested against gram positive strain *S. aureus*. Ciprofloxacin (20 μg/mL) served as standard which produced 23.2 ± 0.4 mm zone of inhibition. Again, maximum zone of inhibition against *E. coli* by Extract, AgNPs, Ag-Extract-NPs were 28.0 ± 0.9 mm, 20.6 ± 0.9, 34.6 ± 0.8 mm respectively. *S. typhi* showed resistant against the tested material as they couldn’t produce remarkable zone of inhibition. Finally, 20.6 ± 0.7 mm, 20.0 ± 0.6 mm, 26.4 ± 0.4 mm zone of inhibition were produced by Extract, AgNPs, Ag-Extract-NPs against *P. aeruginosa*. Ciprofloxacin was used as standard in all cases. In most cases, it was found that Ag-Extract-NPs produced much better zone of inhibition than AgNPs and *M. charantia* extract produced individually. *S. typhi* is more resistant to Ag-Extract-NPs than other gram negative bacteria tested in this study.

**Table 2:** Antibacterial activity of *M. charantia* extract, AgNPs and Ag-Extract-NPs

Items	Zone of inhibition (mm)			
	*S. aureus*	*S. typhi*	*E. coli*	*P. aeruginosa*
	ATCC 25923	ATCC 14028	ATCC 25922	ATCC 27853
*M. charantia* extract (50 μg/ml)	16.6 ± 0.5***	8 ± 0.3***	28.0 ± 0.9*	20.6 ± 0.7***
AgNPs (50 μg/ml)	14.4 ± 0.5***	6.4 ± 0.4***	20.6 ± 0.9***	20.0 ± 0.6***
Ag-Extract-NPs (50 μg/ml)	24.8 ± 0.7***	7.4 ± 0.4***	34.6 ± 0.8***	26.4 ± 0.4*
Ciprofloxacin (20 μg/ml)	23.2 ± 0.4	21.4 ± 0.5	25.6 ± 0.5	25.2 ± 0.4

Mean ± SEM (*n* = 5); Data was analyzed using one way ANOVA followed by Dunnet’s t-test and compared with standard. *p < 0.05, **p < 0.01, ***p < 0.005 were considered significant

The antimicrobial action AgNPs on microorganisms is partially known. Silver nanoparticles possess positive charge which can be attached with negative charged components in cell wall of microorganisms by the electrostatic attraction. Moreover, silver nanoparticles are associated with the thiol groups of cell wall resulted in the generation of reactive oxygen species and destroy the cell wall. In addition, AgNPs can improve the permeability of cell wall by pit formation. As a result, internal materials can leak out and cell may die [5, 22]. On the other hand, fruit extract of *M. charantia* contains of glycosides, saponins, alkaloids, reducing sugars, resins, phenolic constituents, fixed oil and free acids [16]. These phytochemicals can attach with the cell proteins and disrupt their synthesis.

The combine antibacterial efficacy of AgNPs and *M. charantia* (Ag-Extract-NPs) is much better than the effect produced by AgNPs and Extract individually. Perhaps, when the bioactive phytoconstituents of *M. charantia* extract were capped on AgNPs, they produced synergistic effect. As a result, Ag-Extract-NPs can produce strong antibacterial effect.

## Conclusion

In summary, our study shows that Ag-Extract-NPs have excellent antibacterial efficacy both gram positive and negative bacteria. Although, AgNPs and *M. charantia* both possess antimicrobial effect, combination of these two in form of Ag-Extract-NPs showed synergistic effect which is much better than individual effects. This green and safe method may become effective against the resistant bacteria species which is a much concerning issue of medical science now-a-days. However, further investigation is needed on human model to warrant this finding for potential therapeutic effects.

## Additional file

Additional file 1: Table S1. Antibacterial efficacy of *M. charantia*, AgNPs and Ag-Extract-NPs. (XLSX 13 kb)

## Abbreviations

Ag-Extract-NPs: Silver extract nanoparticles
AgNPs: Silver nanoparticles
FTIR: Fourier Transform Infrared Spectroscopy
NPs: Nanoparticles
NSTU: Noakhali Science and Technology University
SEM: Scanning Electron Microscopy
TEM: Transmission Electron Microscopy
UV: Ultraviolet
